# The six-minute walk test in community dwelling elderly: influence of health status.

**DOI:** 10.1186/1471-2318-4-6

**Published:** 2004-07-23

**Authors:** Ivan Bautmans, Margareta Lambert, Tony Mets

**Affiliations:** 1 Gerontology, Free University of Brussels (VUB), Belgium; 2 Geriatrics, Academic Hospital of the Free University of Brussels (VUB), Laarbeeklaan 101, B-1090 Brussels, Belgium

## Abstract

**Background:**

The 6 minutes walk test (6MWT) is a useful assessment instrument for the exercise capacity of elderly persons. The impact of the health status on the 6MWT-distance in elderly, however, remains unclear, reducing its value in clinical settings. The objective of this study was to investigate to what extent the 6MWT-distance in community dwelling elderly is determined by health conditions.

**Methods:**

One hundred and fifty-six community dwelling elderly people (53 male, 103 female) were assessed for health status and performed the 6MWT. After clinical evaluation, electrocardiography and laboratory examination participants were categorized into a stratified six-level classification system according to their health status, going from **A **(completely healthy) to **D **(signs of active disease at the moment of examination).

**Results:**

The mean 6MWT-distance was 603 m (SD = 178). The 6MWT-distance decreased significantly with increasing age (ANOVA p = 0.0001) and with worsening health status (ANCOVA, corrected for age p < 0.001).

A multiple linear regression model with health status, age and gender as independent variables explained 31% of the 6MWT-distance variability. Anthropometrical measures (stature, weight and BMI) did not significantly improve the prediction model. A significant relationship between 6MWT-distance and stature was only present in category A (completely healthy).

**Conclusions:**

Significant differences in 6MWT-distance are observed according to health status in community-dwelling elderly persons. The proposed health categorizing system for elderly people is able to distinguish persons with lower physical exercise capacity and can be useful when advising physical trainers for seniors.

## Background

Aging results in an important decrease of muscle power and exercise capacity[1]. Therefore, elderly often function at the limit of their capacity in order to fulfill the activities of daily living [2]. Determination of the remaining physical capacity can be important in clinical decision-making. From previous studies [3] we observed that one in five elderly patients (70 years and over) is unable to execute the classical treadmill based exercise test, either for fear of falling or because of physical or cognitive limitations. The six-minute walking test (6MWT) is a valid alternative, evaluating the exercise capacity at levels corresponding more to efforts commonly performed by elderly during daily activities.

The 6MWT has first been introduced as a functional exercise test by Lipkin in 1986 [4]. Its results are highly correlated with those of the 12 minutes walk test [5] from which it was derived [6] and with those of cycle ergometer or treadmill based exercise tests [3]. The 6MWT is also a valuable instrument to assess progression of functional exercise capacity in different clinical intervention studies [7-11]. The reliability of the test in healthy elderly persons is high (Intra Class Correlation = 0.93) [12] and it is considered as a valid and reliable test to assess the exercise capacity of elderly patients with chronic heart failure (CHF) and chronic obstructive pulmonary disease (COPD) [3,6,13].

Several authors studied the determining factors of the 6MWT-distance in healthy adults and propose either reference equations or normative data for the 6MWT-outcome [14-16]. Troosters et al. [15] found that age, gender, height and weight explained 66% of the 6MWT-distance variability in 51 healthy adults (free from injury and without history of hospitalization or chronic disease influencing exercise capacity) aged 50–85 years. In another study, Enright et al. [14] administered the 6MWT to 290 healthy persons between 40 and 80 years old (health based upon age<80 years; BMI<35 kg/m^2^; ankle-arm blood pressure index <0.9; 1-second forced expiratory volume and absence of stroke history, use of diuretics and smoking). They report that gender-specific reference equations based upon age, height and weight explained 40% of the variance in 6MWT-distance, which was significantly less for men and women who were older and heavier, and for shorter men. Also, Rikli et al. [16], who measured the 6MWT-distance in respectively 1187 and 2721 community dwelling, functionally independent men and women (who were ambulatory without regular use of assistive device and without medical conditions or physical or cognitive limitations interfering with the test procedures) found a significant 5-year age-group decline in 6MWT-distance (after dividing the study population into gender-specific 5-year age categories) and significantly better scores for men compared to women. However, in a clinical setting one mainly has to deal with elderly having health problems and it can be presumed that their exercise capacity will generally be lower than that of healthy elderly. The available information concerning the impact of health status on the 6MWT-distance in elderly is limited to obesity [17], to the role of muscle strength in persons with mobility limitations [18] and to the influence of reduced aerobic capacity in patients with pulmonary or cardiovascular disease [13,19,20]. Since in current clinical practice geriatricians and physicians are mainly dealing with patients presenting a widespread and heterogeneous variety of co-morbidity, the results of the aforementioned studies offer only limited information concerning the prognostic value and the applicability of the 6MWT in clinical settings different from CHF and COPD. Therefore, we planned this study in order to investigate to what extent the 6MWT-distance in community dwelling elderly is influenced by a broader spectrum of health conditions.

## Methods

### Participants

All members of a large Belgian Health Insurance Organization (BHIO) who registered for a health-conditioning week for seniors, organized by the BHIO, were invited by advertisement to participate in our study. The program of the health-conditioning week included general instructions for a healthy life-style and physical exercise classes. One hundred and fifty-six subjects (53 male, mean age 64.1 years, SD = 6.9; 103 female, mean age 65.5, SD = 7.7) volunteered to participate in the study and gave their informed consent. All participants were living in the community and belonged to the A-category according to Katz et al. [21] and thus were independent for basic activities of daily living.

### Health categories

All participants underwent extensive health screening by medical doctors. First, blood & urine samples were collected after overnight fasting for determination of erythrocyte sedimentation rate, mean red blood cell corpuscular volume, leukocyte count (with differentiation), and concentration of haemoglobin, urea, alkaline phosphatase, glucose, Aspartate Aminotransferase (ASAT), Alanine Aminotransferase (ALAT), protein and electrophoresis for the blood samples and determination of protein, glucose and sediment for the urine samples (according to the SENIEUR protocol [22]). Second, by means of self-administered standardized questionnaires, which were completed by interview, information was obtained regarding medical history, actual diseases, medication use, tobacco and alcohol consumption. Next, all participants underwent physical examination and standard 12-lead electrocardiography (ECG). Based upon this information, participants were then classified into health categories by one of us (TM), before performing the 6MWT. All evaluations were performed on the same day.

The classification system was originally developed in order to grade the participants according to the risk for dangerous complications during physical exercise and to allow physical therapists to adapt the scheduled program of the health-conditioning week (consisting in general instructions for a healthy life-style and physical exercise classes). Therefore, cardiovascular abnormalities were considered to present a higher risk than non-cardiovascular conditions.

We distinguished four categories of decreasing health (see table 1). Subjects categorized as **A **were completely healthy and were considered as presenting no particular risk for any kind of physical exercise. An additional distinction can be made between those using no medication (A1) and those using preventive medication only (A2). This subdivision might be important in specific clinical contexts (e.g. assessment of Vitamin D levels in elderly); in the context of our study the distinction between health categories A1 and A2 is less relevant and therefore these participants will be considered together in all statistical analysis. Category **B **consisted of participants who were functioning normally, presented no major medical restrictions, but could be in need of special instructions for exercising due to their health status. Category **B1 **was accorded to participants having a disease that was non-cardiovascular and stable. Category **B2 **was given to participants using medication having cardiovascular effects. Subjects in category **C **had cardiovascular pathology or a history thereof; they were considered as having an increased risk of cardiovascular complications during exercise. Those belonging to category **D **were found to present signs of acute disease or exacerbation of chronic disease. If combinations of health conditions existed, subjects were classified in the worst health category.

**Table 1:** Health categories

**Health Category**	Description *	**Clinical examples**
A A1	Completely healthy; no medication	
A2	Completely healthy; using only preventive medication	Hormonal replacement therapy, aspirin, ...
B B1	Functioning normally; presence of stabilised, non cardiovascular disease; absence of cardiovascular abnormalities	treated hypothyroidism, stable diabetes, ...
B2	Functioning normally; using medication with cardiovascular effect, no overt cardiovascular disease other than normalized arterial hypertension	Arterial hypertension; β blocking agent, ...
C	(history of) cardio-vascular pathology or abnormal ECG.	Bundle branch block; angina, CABG; ...
D	Presenting signs of acute or active disease at the moment of examination.	bronchospasm, swollen joints, influenza, ...

* Status after questioning, physical examination, ECG, and laboratory examination of blood, serum & urine according to the SENIEUR protocol [22]. CABG: coronary artery bypass graft

### Measurements

Subjects were assessed for weight, height and body mass index [BMI = weight / (height)^2^]. Before starting the health conditioning week, all participants performed the 6MWT following a protocol as previously described [3]. All participants were naive to the 6MWT. Each participant was tested individually and was constantly observed by a physical therapist, who was unaware of the health category attribution. The 6MWT was performed outdoors upon a hardened and flat surface following a circular circuit of 121 m. Participants were instructed to try to cover as much distance as possible within six minutes without running. They wore comfortable shoes and clothing and were allowed to rest or stop when necessary.

### Statistical analysis

Statistical analysis was performed using the SPSS (for Windows, release 11.5.1) software package. All data subsets were assessed for the presence of a normal distribution (Kolmogorov-Smirnov Goodness of Fit Test p > 0.05) before using parametric analysis. Correlations between data subsets with a normal distribution were performed using Pearson's Correlation Coefficient; non-normally distributed datasets were analyzed for correlation using Kendall's Correlation Coefficient. Differences between data subsets were analyzed using Analysis of Variance (ANOVA), Analysis of Co-Variance (ANCOVA) and Students t-test. Bonferroni post-hoc test was performed to detect significant differences between subgroups. A multiple linear regression model was designed in order to explain the variability of the 6MWT-distance. Significance level was set at p < 0.05 [23].

## Results

The mean 6MWT-distance was 603 m (SD = 178) for the whole group (N = 156). Overall, male participants were significantly heavier (p < 0.001), taller (p < 0.001) and covered a significantly longer distance in six minutes (p = 0.022) than female participants. There was no significant difference in age or BMI between male and female participants (table 2).

**Table 2:** Participants' characteristics.

	**Male N = 53** **mean (SD)**	**Female N = 103** **mean (SD)**
6MWT (m)	647.9 (201.8)	579.3 * (159.8)
Age (years)	64.1 (6.9)	65.5 (7.7)
Weight (kg)	80.5 (11.0)	69.4^† ^(11.4)
Height (cm)	171.3 (6.6)	158.2^† ^(6.3)
BMI (kg/m^2^)	27.4 (3.1)	27.8 (4.2)

*p < 0.05, ^†^p < 0.001

**Figure 1:**
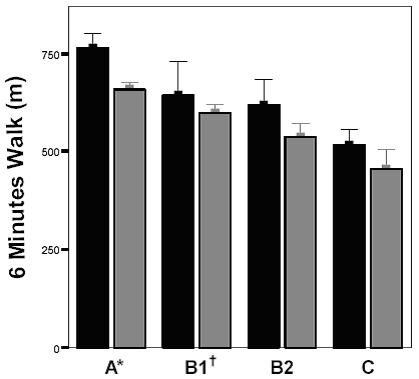
**Impact of Health Status on 6-Minute Walk Distance in Community Dwelling Elderly. **■ Male Participants N = 53.  Female Participants N = 102. Bars represent mean values ± SE. Significant decrease of 6 Minutes Walk Distance with worsening health category (ANCOVA corrected for age p < 0.01). * Significantly higher 6 MWT-distance than categories **B2**and **C** (Bonferroni post-hoc test p < 0.01) ^†^Significantly higher 6 MWT-distance than category **C** (Bonferroni post-hoc test p < 0.05).

Since there was only one participant in health-category **D**, this subgroup was excluded from all statistic tests. Fifty-eight (37%) participants (44 in group **A1 **and 14 in group **A2**) could be considered as completely healthy. No significant difference was found regarding age or 6MWT-distance between participants of group **A1 **and **A2 **(mean age respectively 62.0 years SD = 6.6 and 62.7 years SD = 10.0, mean 6MWT-distance respectively 696.4 m SD = 150.7 and 686.9 m SD = 118.4 for **A1 **and **A2**). ANOVA testing (male and female considered together) revealed a significantly lower 6MWT-distance (p < 0.001) with worsening health category (**A **→ **C**), even after correction for age (ANCOVA p < 0.001). Participants with poorer health status were significantly older (p = 0.0001) than those in better health (Figure 1 and Table 3).

**Table 3:** Characteristics of health groups.

**Category**	**N**	**Male/Female**	**Age [years] **mean (SD)
A	58	20/38	62.2 *, ^†^(6.7)
B1	30	7/23	63.5 * (6.6)
B2	35	11/24	69.0 (6.6)
C	32	15/17	67.2 (6.6)
D	1	0/1	73
All	156	53/103	65.1 (7.4)

* Significantly younger than category. **B**_2 _(Bonferroni post-hoc test p < 0.01) ^†^Significantly younger than category. **C **(Bonferroni post-hoc test p < 0.01)

Post-hoc tests allowed detecting significant differences in the 6MWT-distance between health-categories **A **&**B2**(p < 0.01), **A **&**C **(p < 0.01), and **B1 **&**C **(p < 0.05). Subjects in health-category **A **were significantly (p < 0.01) younger than those in category **B2 **and **C**; those in health-category **B1 **were significantly (p < 0.01) younger than category **B2**.

There was a negative correlation between the 6MWT-distance and the participant's age for the whole group (r = -0.42, p < 0.001), and for both males (r = -0.32, p = 0.019) and females (r = -0.47, p < 0.001). A correlation between 6MWT-distance and age was present within health-categories **A **(r = -0.38; p = 0.004), and **C **(r = -0.40; p = 0.023), but not within category **B1 **(r = -0.21; p = 0.269) or **B2 **(r = -0.27; p = 0.121) (table 4). No statistically significant correlations were found between 6MWT-distance and weight or BMI for the whole group or within the different health status categories. Height was significantly correlated with 6MWT-distance (r = 0.33, p = 0.012, Table 4) only in the group of completely healthy participants (category **A**).

**Table 4:** Relationships between 6MWT-distance and participants' characteristics.

**Category**	**Age**	**Weight**	**Height**	**BMI**
All (N = 156)	-0.42*	-0.03	0.13	-0.14
A (N = 58)	-0.38^†^	0.10	0.33*	-0.20
B1 (N = 30)	-0.21	0.07	0.02	0.05
B2 (N = 35)	-0.27	-0.10	-0.01	-0.10
C (N = 32)	-0.40*	-0.14	0.08	-0.29
Male	-0.32*	-0.19	-0.24	-0.07
Female	-0.47*	-0.09	0.15	-0.18

Values represent correlation coefficients. *p 0.05, ^†^p < 0.01

**Figure 2:**
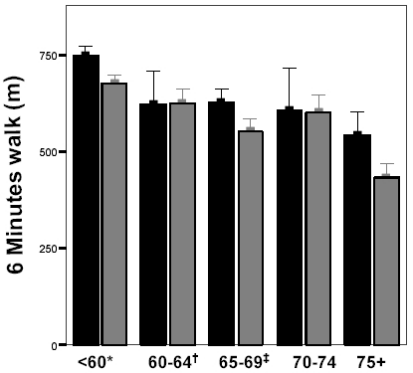
**Impact of Age on 6-Minute Walk Distance in Community Dwelling Elderly. **■ Male Participants N = 53.  Female Participants N = 102. Bars represent mean values ± SE. Significant decrease of 6 Minutes Walk (6 MWT) Distance with increasing age category (ANOVA p < 0.01). * Significantly higher 6 MWT-distance than categories 65–69 and 75+ (Bonferroni post-hoc test p < 0.05 and p < 0.01 respectively).  Significantly higher 6 MWT-distance than category 75+ (Bonferroni post-hoc test p < 0.01). ^‡^Significantly higher 6 MWT-distance than category 75+ (Bonferroni post-hoc test p < 0.05).

When the 156 participants were divided in age categories, again a significant (p < 0.001) decrease in 6MWT-distance was observed with increasing age (figure 2). Subjects aged 75 years and more covered a significantly (p < 0.05) shorter distance in the 6MWT than those less than 65 years of age, regardless of their health status. Two-way ANOVA revealed no significant interaction between gender, health status and age concerning the 6MWT-distance (table 5).

**Table 5:** Two-way ANOVA for 6MWT-distance

**Source of Variation**	**F-value**	**P-value**
Health-category by Age-category	0.65	0.833
Health-category by Gender	0.20	0.937
Age-category by gender	0.40	0.807

**Figure 3:**
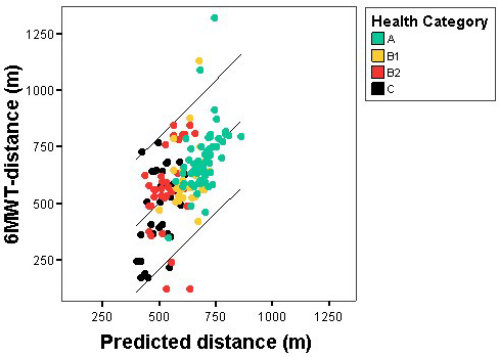
**Predicted and Actual 6-Minute Walk Distance in Community Dwelling Elderly. **The bullets represent individual values according to the attributed health category: Green bullets Health Category **A, **Yellow bullets Health Category **B1, **Red bullets Health Category **B2 **and Black bullets Health Category **C. **The lines represent the fit line and the 95% confidence interval. The predicted values are based upon the proposed multiple linear regression model 6MWT-distance predicted (m) = 1192 - (6 × age) - (57 × health category) -(69 × gender) with age expressed in years; 0 for male and 1 for female; 1 for health-category **A**, 2 for health-category **B1**, 3 for health-category **B2**, 4 for health-category **C**. The standard error of the estimate is 147 m.

In a first step a multiple linear regression model was computed with 6MWT-distance as dependent variable and with age, gender, weight, height and health status as independent variables. Analysis of this regression model (R^2 ^= 0.33) revealed that neither weight nor height was a significant coefficient, contributing to the model (t = -1.4, p = 0.16 and t = -0.98, p = 0.33 for weight and height respectively). Therefore, a new multiple linear regression model was computed without weight and height as independent variables explaining 31% of the variability of the 6MWT-distance (R^2 ^= 0.31). In figure 3 the measured individual 6MWT-distances are plotted against the predicted distances based upon the following regression equation:

6MWT-distance predicted (m) = 1192 - (6 × age) - (57 × health category) - (69 × gender)

with age expressed in years; 0 for male and 1 for female; 1 for health-category **A**, 2 for health-category **B1**, 3 for health-category **B2**, 4 for health-category **C**. The standard error of the estimate was 147 m.

In order to determine the impact of health status in the regression model, we have computed a model with 6MWT-distance as dependent variable and with age and gender as independent variables (R^2 ^= 0.18) in a first step, in which we introduced in a second step the entry of health status as supplementary independent variable. Using this method we obtained the partial correlation coefficient (partial r = -0.40, p < 0.001) between health status and 6MWT-distance.

## Discussion

The 6MWT presents several interesting advantages for the evaluation of the exercise capacity in elderly people. Different authors have described reference equations and tables to predict the 6MWT-distance in healthy elderly subjects. Gender, age, weight and height of these subjects appear to explain a large proportion of the variability in the 6MWT-distance [14-16]. Advancing age, however, is predominantly accompanied by an increasing burden of pathology [24] and "apparently healthy" elderly persons, willing to participate in physical training sessions, actually present a large diversity in health status. This means that the exercise capacity and the risk for complications during exercise are not the same for each person who consider themselves able to perform physical exercise. Ideally, an exercise program must be established for each person individually considering all facets of his/her health condition. It is conceivable that healthier elderly will present a better physical exercise capacity than those with a worse health condition. Therefore it is to be expected that the performance on exercise tests will reflect the aforementioned difference in health status. However, the contribution of the health status of elderly participants to the variability of the distance walked in the 6MWT has not been extensively described. In order to distinguish completely healthy participants from those with a worse health status we used criteria derived from the SENIEUR protocol [22]. This protocol was originally established to select participants for studies concerning the age-related changes of the immune system and allows the distinction between age-related and disease-related changes. The literature provides no clear guidelines to categorize elderly persons attending exercise programs according to their health status. Therefore, we developed a classification system, which stratifies health categories corresponding to an increasing risk for complications during physical exercise. Since the SENIEUR protocol allows for the presence of certain diseases and the use of medication that has no influence on the immune system, in our classification these criteria were further adapted in order to identify the "completely healthy" participants (group **A**) and to differentiate them from the "apparently healthy". The latter group was functioning normally, but could not be considered as completely healthy since participants either presented stabilized non-cardiovascular conditions (group **B1**) or were using cardiovascular medication, however, without any sign of active cardiovascular disease other than normalized hypertension and without significant abnormalities on ECG (group **B2**). When a history or signs of cardiovascular disease, other than hypertension, were noticed, participants were considered as belonging to group **C**. When evaluating large numbers of participants there is always the possibility of encountering individuals who are acutely ill; since they will not be able to fully participate in an exercise program, we choose to classify them separately (group **D**). The classification system was primarily designed to allow the establishment of recommendations concerning the exercise schedule (type, duration and intensity) of elderly persons in the absence of direct medical supervision. Therefore, the classification system is rather conservative and it is easy for an individual to be considered at risk for complications. Roughly, participants in category **A **will have no particular limitations for exercising; for those in category **B1 **the instructions will vary with the nature of the health problem; those in category **B2 **will only exercise at higher intensity (e.g. up to 80% of maximal heart rate or higher) when guided by an instructor qualified for training elderly persons; those in category **C **will only be allowed to exercise under supervision of an instructor and with medical guidance of the training program; those in category **D **will not exercise unless cleared by a physician. Since any exercise schedule will depend upon its objective, we do not go into further details here for these recommendations.

It is clear that elderly persons often termed as 'apparently healthy' do not correspond to criteria for being completely healthy. In our study, only 28% (group **A1 **= 44 of 156) or 37% (Groups **A1 **and **A2 **= 58 of 156) of the participants were completely healthy, depending on whether the use of preventive medication was taken into account. However, 79% (groups **A**, **B1 **and **B2 **= 123 of 156) were functioning normally in the community and had no overt history of cardiovascular disease. Rikli et al. (1999) [16] studied the 6MWT-distance in 7183 community-dwelling older adults (5048 male, 2135 female) aged 60–94 years. According to the inclusion criteria they describe, we can assume that the participants of that study meet our classification criteria of categories **A**, **B1 **and **B2**. Our study, based upon a much smaller population sample, demonstrates that normative data based upon the population of the aforementioned authors is not representative for completely healthy elderly. Indeed, the mean 6MWT-distance of completely healthy participants (category **A**, mean age 62 years (SD = 7); mean 6MWT-distance 764 m (SD = 162) and 657 m (SD = 118) for male and female respectively) is much higher than the norm reported by Rikli et al. [16] for subjects in the same age category (60–64 years; mean 6MWT-distance 616 m (SD = 84) and 551 m (SD = 77) for male and female respectively). When age-matched categories with mixed health status are compared, our results (see figure 2) accord well with those of Rikli et al. [16]. Neither the studies of Enright et al. [14] or Troosters et al. [15] used the criteria of the SENIEUR protocol to select healthy individuals. This probably means that the health status of the populations they describe is heterogeneous and might also explain the considerable range in 6MWT-distance (383–820 m) encountered in the 'healthy' elderly (50–85 years old) participants of Troosters et al. [15] as well as the relatively low median 6MWT-distances (576 m and 494 m for male and female respectively) for 'healthy' adults (40–80 years old) in the study of Enright et al. [14]. Our study demonstrates that the proposed health-categorization is able to detect a significant reduction in physical capacity due to individual parameters such as medical history, medication use and current health status, other than age, gender (explaining only 18% of the variability) and anthropometrical parameters (no significant independent predictors when health status is included in the regression model). It is our opinion that the results of our study argue in favor of the validity of the proposed health categorization. At this moment, however, other data concerning the reliability of the classification system are not yet available. The results of the multiple linear regression analysis and the absence of a significant interaction (two-way ANOVA testing) between health status, age and gender confirm that each of these factors is an independent determinant of 6MWT-distance in the elderly. The proposed health categorization is able to discriminate the completely healthy elderly from the apparently healthy and to distinguish among the apparently healthy several categories. This results in a more diversified spectrum of the community dwelling elderly population than obtained with other categorizations like the New York Heart Association (NYHA)-classification for cardiovascular disease [25] and the American Heart Association (AHA) risk stratification criteria [26]. Currently, these systems are widely used for stratification purposes in physical training schedules. However, they were developed for the description of cardiac patients and do not consider other pathologies nor co-morbidities.

A minor familiarization effect has been described for the 6MWT with better values obtained at a second testing [27]. Since repeated testing is less applicable in clinical settings, we chose to use the results from a single test administration when the participants were still naïve for the 6MWT. In our study the correlation of the 6MWT-distance with age, gender and stature as described by others [14-16] is confirmed in the group of the completely healthy participants (category **A**). This relationship is attenuated or disappears in subjects presenting chronic pathology even without apparent functional limitations. Our study demonstrates that there exists a significant decrease in 6MWT-distance with increasing health problems. Since the main objective of our classification system was to stratify for the risk for cardiovascular or metabolic (e.g. hypoglycemia) complications during exercise, we did not expect that the performance on the 6MWT would vary with the health categories. It is probable that the relationship between health status and 6MWT-distance reflects the influence of the status of the cardiovascular system on the general fitness. Inversely, however, it can also be explained by the negative prognostic value of lack of fitness for the ulterior development of cardiovascular disease. It has, indeed, repeatedly been reported for healthy middle-aged adults that this risk is greater for the least fit individuals [28,29]. Since most of our participants were older than those in the risk studies, a certain amount of cardiovascular pathology did occur.

In a multiple linear regression model age, gender and health status explained 31% of the variability of the 6MWT-distance in the population of our study. All three independent variables had a highly significant contribution to the model (age and gender together accounted for 18% of the variability, partial correlation between health status and 6MWT-distance = -0.40, p < 0.001). The addition of weight, height and BMI to the prediction model did not significantly improve the R^2^-value. This is different from other studies that describe regression equations for the 6MWT-distance in healthy elderly [14,15], and indicates that the health condition of community dwelling elderly is more influencing the 6MWT-distance than their anthropometrical predisposition (weight and height are not significant independent predictors for 6MWT-distance after considering health status, age and gender). The fact that these authors obtained a much higher R^2^-value (R^2 ^= 0.66 [15], R^2 ^= 0.42 for male and R^2 ^= 0.38 for female [14]) than ours (R^2 ^= 0.31) can be due to the more heterogeneous composition in health status and the repartition of the population in several health categories in our study. The remaining unexplained variability in 6MWT-distance might be found in the differences in skeletal muscle strength, the training levels and the physical activity levels of the participants. However, these parameters were not measured in our study.

We propose to incorporate the present health classification for elderly people as outcome of the medical screenings preceding the start of a physical exercise program. As shown in our study, significant differences can be found in physical exercise capacity according to the health status. We used the 6MWT-distance, which is an established exercise test for elderly persons, to document the physical exercise capacity of our participants. Especially in the elderly, which show increased prevalence of co-morbidities (most frequently (cardio)vascular, metabolic or rheumatological), physical exercise programs should be established according to the individual exercise capacity. Following the health classification of the elderly participant, the training instructor can adapt the modalities of the training program in order to obtain the best results at the most appropriate training intensity. This should finally lead to concrete training guidelines for elderly people in relation to their health status, diminishing the risk for injuries or complications during physical exercise.

## Competing interests

None declared.

## Authors' contributions

TM conceived and coordinated the study, participated in the evaluation of the health condition of the participants, the analysis and the redaction. IB performed the statistical analysis and participated in the design and the redaction. ML participated in the evaluation of the health condition of the participants, the analysis and the redaction. All authors read and approved the final manuscript.

## Pre-publication history

The pre-publication history for this paper can be accessed here:


